# Simulating cyanobacterial phenotypes by integrating flux balance analysis, kinetics, and a light distribution function

**DOI:** 10.1186/s12934-015-0396-0

**Published:** 2015-12-24

**Authors:** Lian He, Stephen G. Wu, Ni Wan, Adrienne C. Reding, Yinjie J. Tang

**Affiliations:** 1 Department of Energy, Environmental and Chemical Engineering, Washington University, St. Louis, MO 63130 USA; 2 Department of Mechanical Engineering and Materials Science, Washington University, St. Louis, MO 63130 USA; 3 Department of Biochemistry and Molecular Biology, College of Wooster, Wooster, OH 44691 USA

**Keywords:** Glycogen, Multiple-scale modeling, Photobioreactors, Photosynthesis efficiency, Self-shading, *Synechocystis* 6803

## Abstract

**Background:**

Genome-scale models (GSMs) are widely used to predict cyanobacterial phenotypes in photobioreactors (PBRs). However, stoichiometric GSMs mainly focus on fluxome that result in maximal yields. Cyanobacterial metabolism is controlled by both intracellular enzymes and photobioreactor conditions. To connect both intracellular and extracellular information and achieve a better understanding of PBRs productivities, this study integrates a genome-scale metabolic model of *Synechocystis* 6803 with growth kinetics, cell movements, and a light distribution function. The hybrid platform not only maps flux dynamics in cells of sub-populations but also predicts overall production titer and rate in PBRs.

**Results:**

Analysis of the integrated GSM demonstrates several results. First, cyanobacteria are capable of reaching high biomass concentration (>20 g/L in 21 days) in PBRs without light and CO_2_ mass transfer limitations. Second, fluxome in a single cyanobacterium may show stochastic changes due to random cell movements in PBRs. Third, insufficient light due to cell self-shading can activate the oxidative pentose phosphate pathway in subpopulation cells. Fourth, the model indicates that the removal of glycogen synthesis pathway may not improve cyanobacterial bio-production in large-size PBRs, because glycogen can support cell growth in the dark zones. Based on experimental data, the integrated GSM estimates that *Synechocystis* 6803 in shake flask conditions has a photosynthesis efficiency of ~2.7 %.

**Conclusions:**

The multiple-scale integrated GSM, which examines both intracellular and extracellular domains, can be used to predict production yield/rate/titer in large-size PBRs. More importantly, genetic engineering strategies predicted by a traditional GSM may work well only in optimal growth conditions. In contrast, the integrated GSM may reveal mutant physiologies in diverse bioreactor conditions, leading to the design of robust strains with high chances of success in industrial settings.

**Electronic supplementary material:**

The online version of this article (doi:10.1186/s12934-015-0396-0) contains supplementary material, which is available to authorized users.

## Background

In photobioreactors (PBRs), light penetration depth at high cell density can be as short as a few centimeters [1]. Thus, during large-size PBR cultivation, cyanobacteria move continuously between the “light zone” (where light is sufficient) and the “dark zone” (where light is substantially shaded). As a consequence, cyanobacterial metabolism in PBRs is spatially and temporally dependent: cells have autotrophic growth in the light zone, and they perform heterotrophic growth in the dark zone by consuming energy-storage compounds. Moreover, PBR performances are also affected by the efficiency of CO_2_ gas–liquid transfer. To enhance mass transfer, people often use CO_2_-enriched air in combination with high intensity mixing. Many models have been developed to understand how cyanobacterial physiological dynamics are influenced by the light intensity, CO_2_ supply, temperature, and geometry of PBRs [2–6]. Those kinetic and reactor studies are useful in optimizing PBR design and operations. However, bioprocess modeling is unable to provide an understanding of intracellular enzyme functions and metabolic fluxes in cyanobacteria. To improve engineered microalgae strains’ metabolisms in large-size PBRs, it is necessary to link process models to metabolic models.

On the other hand, metabolic flux analyses (MFA) can quantify in vivo enzyme reaction rates, and thus allow us to investigate the flux phenotypes resulting from complicated gene-protein-metabolite regulations. ^13^C-MFA measures carbon fluxes through the central metabolism via ^13^C labeling experiments. Alternatively, genome-scale flux balance analysis (FBA) can generate a holistic intracellular flux distribution map [7] owing to its extended coverage of genomic information [8]. Computational platforms, such as COBRA [9] and OptForce [10], can predict genetic targets and guide rational designs of engineered strains. FBA can also be integrated with constraint-based elementary flux mode analysis to identify optimal pathways for bio-productions [11]. However, an inherent limitation of traditional GSM is that it predicts only flux distributions that result in maximal yields in an optimal culture condition. They cannot forecast mutant strains’ production titers and rates in dynamic and heterogeneous bioreactors.

**Fig. 1:**
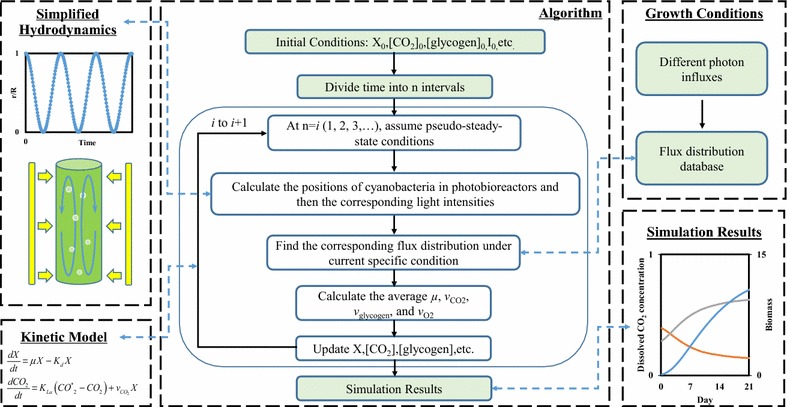
Algorithm for simulating cyanobacterial growth and intracellular flux distribution by integrating the flux analysis model, kinetics, and a light distribution function

In this study, the major goal is to demonstrate multiple-scale modeling approaches by linking cell metabolisms to PBR environmental fluctuations. Specifically, the modeling efforts focus on *Synechocystis* 6803, a most widely used cyanobacterial biorefinery. Appealing traits of this species include amenability to genetic modifications, well-studied genomics, and native genes for biosynthesis of alkanes/alkenes and hydrogen [12–14]. To predict cyanobacterial growth and metabolic flux phenotypes in PBR settings, we integrated a genome-scale cyanobacteria model, iJN678 [15], with growth kinetics, cell movements based on reported PBR hydrodynamics, and a heterogeneous light distribution (Fig. 1). The model assumption is that heterogeneous PBR conditions affect cyanobacteria, leading to heterogeneous cell metabolisms in different sub-populations. Such an approach can provide biological information ranging from the intracellular domain to the PBR domain, and fill the gaps between systems biology and the PBR process. The multiple-scale modeling is useful for estimating mutant strains’ potentials to achieve the production metrics required for commercialization.

## Results

### Simulation of cyanobacterial optimal growth in a cylindrical PBR

**Fig. 2:**
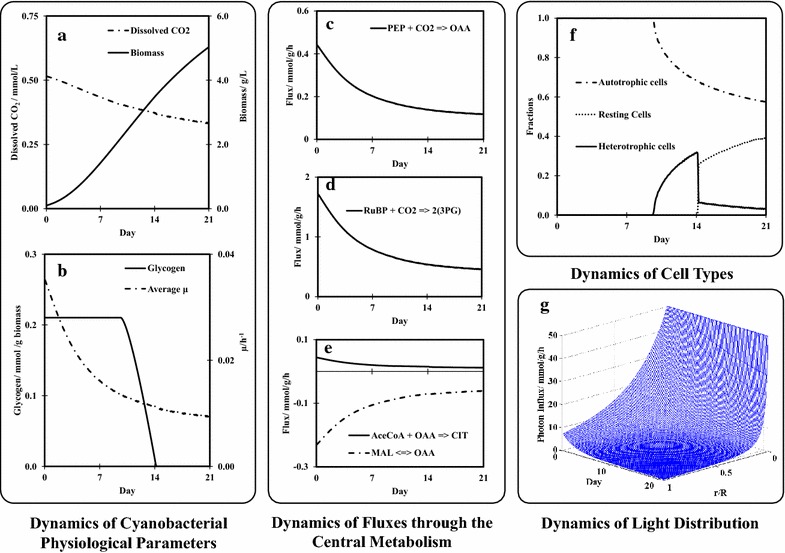
Simulations of dynamics of cyanobacterial performance in a 60 mm-radius cylindrical PBR under 50 *µ*E/m^2^/s surface light intensity. **a** biomass concentration and dissolved CO_2_/HCO_3_^−^ concentration; **b** glycogen content in biomass and average specific growth rate; **c** flux through the PEP carboxylase reaction; **d** flux through the RuBP carboxylase reaction; **e** fluxes through the TCA cycle (negative flux means the flux direction is reversed); **f** ratios of autotrophic, heterotrophic, and resting sub-populations; and **g** light distribution in the bioreactor as a function of time and relative distance r/R, where r is the local position and R is the radius of the bioreactor. The parameters used for simulation are given in Table 1. Abbreviations of metabolites: *3PG* 3-phosphoglycerate, *AceCoA* acetyl-CoA, *CIT* citrate, *MAL* malate, *OAA* oxaloacetate, *PEP* phosphoenolpyruvate, and *RuBP* ribulose-1,5-bisphosphate

The integrated GSM was first applied to predict cyanobacterial growth in a cylindrical PBR, which was assumed to have a radius of 60 mm and a constant surface light intensity of 50 *µ*E/m^2^/s. Although the maximal photosynthetic efficiency in photosynthetic species can reach 4.6–6 % [16], not all incident radiation in PBRs can be efficiently used by cyanobacteria, thereby resulting in a lower conversion efficiency [17]. Hence, we chose a photosynthesis efficiency of 1.5 %, which was within a reasonable range of actual photosynthesis efficiencies of microalgae [18]. Based on a previous study, the mass transfer rate of CO_2_ was assumed to be 10 h^−1^ [19]. Under such a condition, cyanobacterial biomass concentration could increase from 0.1 to 5 g/L in 3 weeks, provided that other mineral nutrients are supplied continuously (Fig. 2a and Additional file 2: Fig. S1). The modelling results also showed continuous decreases in the growth rate (Fig. 2b) and intracellular fluxes in the central metabolism (Fig. 2c–e), which was caused by a continuous decrease in local light intensity over time (Fig. 2g). As the ‘dark zone’ expanded, some cyanobacteria switched from autotrophic growth to heterotrophic growth in the late growth phase, and eventually became resting cells (Fig. 2f). The expanding ‘dark zone’ also led to a gradual reduction in glycogen content per gram of biomass, which was the same when all the cells were located in the light zone (Fig. 2b). This prediction agrees with two previous studies [20, 21].

**Fig. 3:**
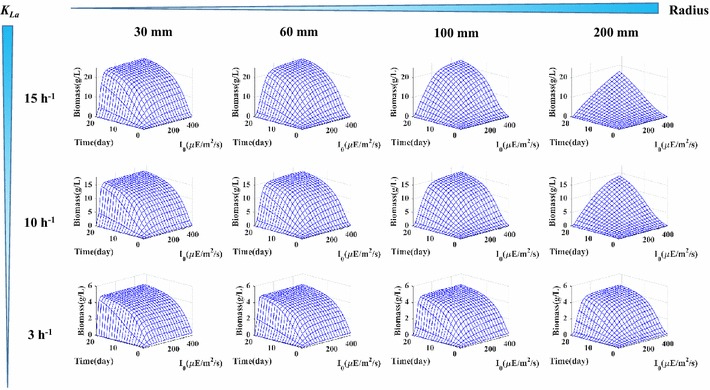
Test of biomass growth performance sensitivity to the mass transfer rate (*K*_*La*_ in h^−1^), surface light intensity (*I*_*0*_ in *µ*E/m^2^/s), and the bioreactor geometry (*R* in mm). Each *three-dimensional figure* shows biomass growth as a function of time and surface light intensity

Next, we tested the sensitivity of biomass production to the mass transfer rate, PBR surface light intensity, and PBR diameter (Fig. 3) . With a light intensity of 100 *µ*E/m^2^/s and a moderate mass transfer rate of 15 h^−1^, small PBRs (30 mm radius) could produce 20 g/L of biomass in 21 days. Although such productivity has been experimentally observed in small PBRs [22], it can be hardly achieved in large-size PBRs. As shown by the model, the biomass productivity is highly sensitive to the surface-to-volume ratios of the PBRs, and increasing the PBR diameter reduces biomass productivity dramatically. Hence, to improve biomass production in PBRs, one needs to reduce the surface-to-volume ratios, increase the culture mixing and air flow rate [23], and maintain a sufficient surface light intensity.

Finally, the simulations also demonstrate that, due to random cell movements in PBRs, single cell fluxome may show stochastic changes (Additional file 2: Fig. S2 b, c). Additionally, we tested the sensitivity of biomass growth to circulation time. The model indicates that perturbing the circulation speeds of cell subpopulations did not affect total biomass production by PBRs, provided that the CO_2_ mass transfer and surface light were constant (Additional file 2: Fig. S3).

### Simulation of cyanobacterial oxidative pentose phosphate pathway in a cylindrical PBR

**Fig. 4:**
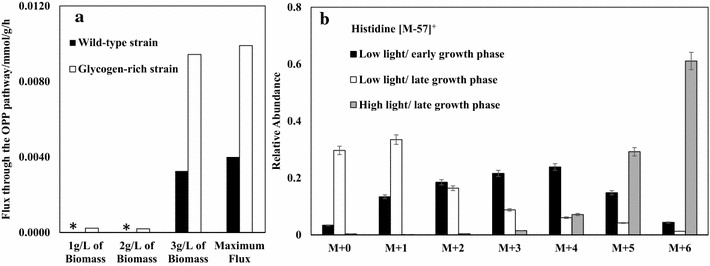
The oxidative pentose phosphate (OPP) pathway in cyanobacteria. **a** Comparison of simulated fluxes through the OPP pathway between wild-type (*black bars*) and glycogen-rich (*white bars*) cyanobacteria strains at different biomass concentrations. ‘*Asterisk*’ means the flux is zero. Compared to the wild type strain, the model assumes that the glycogen-rich strain accumulates five times more glycogen during autotrophic growth and consumes glycogen five times faster during heterotrophic growth. **b** Relative abundance of histidine labeling profile under different *Synechocystis* growth phases. *Black bars* (low light/early growth phase): light intensity of ~50 *µ*E/m^2^/s; *white bars* (low light/late growth phase): light intensity of ~50 *µ*E/m^2^/s; *grey bars* (high light/late growth phase): light intensity of ~100 *µ*E/m^2^/s

In cyanobacteria, the oxidative pentose phosphate (OPP) pathway and the Calvin cycle operates in opposite directions: The former generates CO_2_ and NADPH, while the latter consumes CO_2_ and NADPH. Figure 2d shows that the Calvin cycle had a strong flux in the early growth phase, while the OPP pathway remained silent under light-sufficient conditions (Fig. 4a). In the late growth stage, active fluxes through the OPP pathway appeared (Fig. 4a) due to the self-shading effect. The activity of the OPP pathway increased concurrently with the glycogen consumption rate in darkness (Fig. 4a and Additional file 2: Fig. S4). Thus, an active OPP flux in photoautotrophic cultures is the metabolic response to light deficiency in PBRs. Recent ^13^C-flux measurements also showed positive OPP fluxes in *Synechocystis* 6803 PBR cultures [24, 25]. To further confirm our model predictions, we examined the labelling patterns of histidine by growing *Synechocystis* 6803 with NaH^13^CO_3_ and [1-^13^C] glucose. When glucose was metabolized via the OPP pathway, non-labeled ribose-5-phosphate was generated from [1-^13^C] glucose [26], which is a precursor to histidine. Therefore, an active OPP pathway was expected to reduce the ^13^C-enrichment of proteinogenic histidine. Figure 3b shows that the ^12^C-concentration of histidine was high under low light conditions, supporting the model prediction that light deficiency leads to an active OPP pathway for C6 sugar utilizations.

### Investigation of cyanobacterial photosynthesis efficiency in shake flasks

**Fig. 5:**
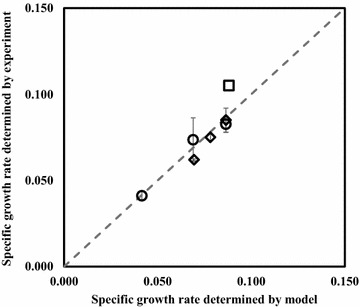
Comparison of experimental and simulation results of cyanobacterial growth rates in shake flasks. *Diamond* markers represent cultures with different volumes under the same light intensity of ~50 *μ*E/m^2^/s. The volumes are 50, 100, and 150 mL, respectively, corresponding to *diamond* markers from *top* to *bottom.* *Circle* markers represent 15 mL cultures growing under different light conditions. The light intensities are ~15, ~25 and ~35 *μ*E/m^2^/s, respectively, corresponding to *circle* markers from *top* to *bottom*. The specific growth rates were calculated based on OD_730_ values in the early exponential growth phase. The *square* marker represents a reported cyanobacterial growth rate in a mini-PBR [27]. The parameters used for simulating cyanobacterial growth are given in Additional file 2: Table S1

Next, we used the integrated GSM to determine the photosynthesis efficiency of *Synechocystis* 6803 by minimizing the sum of squared errors between experimental and simulated averaged specific grow rates. We simplified the geometry of the shake flasks into a two-dimensional rectangle (Additional file 2: Fig. S5), and made the local light intensity dependent on the vertical distance from a cell to the light source. The CO_2_ mass transfer rates in shake flasks were calculated based on Eq. (9). As a consequence, a photosynthesis efficiency of 2.7 % (Additional file 2: Fig. S6) resulted from the best fit of specific growth rates under shake flask cultures (Diamond and circle markers in Fig. 5). Furthermore, this photosynthesis efficiency was used to simulate the growth of *Synechococcus elongatus* UTEX 2973 (a fast-growing cyanobacterium species) in a column PBR (with 3 % CO_2_ and under 500 *µ*E/m^2^/s light intensity) [27]. The model predicted slightly lower specific growth rate than the experimental value (Square marker in Additional file 2: Fig. S5). This difference is possibly due to an increased photosynthesis activity under high CO_2_ concentrations [28].

### Model-based investigation of lactate production by engineered cyanobacteria in PBRs

**Fig. 6:**
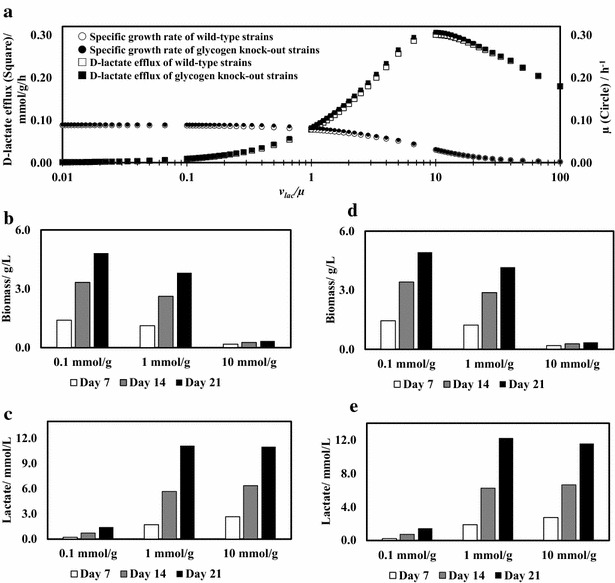
Simulation results of d-lactate producing cyanobacteria performance. **a** FBA simulations of d-lactate flux and growth rate as functions of *v*_lac_/*µ* (mmol lactate/g biomass). *White* *markers* wild-type strain, *black markers* glycogen-knockout strain. **b**, **c** Simulation of biomass growth (**b**) and d-lactate production (**c**) of wild-type cyanobacteria at different *v*_lac_/*µ* ratios in a cylindrical PBR. **d**, **e** Simulation of biomass growth (**d**) and d-lactate production (**e**) of glycogen-knockout cyanobacteria at different *v*_lac_/*µ* ratios in a cylindrical PBR

We further applied the integrated GSM to predict the growth and volumetric d-lactate productivity of engineered cyanobacterial strains, in which a mutated glycerol dehydrogenase was overexpressed for producing optically pure d-lactate [29]. The MOMA algorithm was applied to simulate the metabolism in engineered strains (See Methods). Growth-associated lactate production was assumed (i.e., lactate production was proportional to biomass synthesis). First, we tested the relationship between lactate efflux (*v*_*lac*_) and specific growth rate (*µ*) using only the FBA model. Figure 6a shows *v*_*lac*_ and *µ* as functions of the ratio *v*_*lac*_/*µ,* which denotes the amount of lactate produced per gram of biomass (or mmol lactate/g biomass). Within a wide *v*_*lac*_/*µ* range, from 0.01 to 100 mmol lactate/g biomass, *µ* decreased with increasing *v*_*lac*_/*µ*, but *v*_*lac*_ showed a parabolic tendency, peaking at 0.3 mmol/g/h (Fig. 6a). Next, we used the integrated GSM to simulate the cyanobacterial growth and D-lactate production in PBRs at different *v*_*lac*_/*µ* ratios (Fig. 6b, c). As a result, increasing the *v*_*lac*_/*µ* ratio led to lower biomass production, which, however, did not necessarily improve the overall d-lactate production. For example, when *v*_*lac*_/*µ* was increased from 1 to 10 mmol/g, lactate production in PBRs remained the same, but biomass production was significantly diminished. Enhancing the lactate pathway (i.e., increase *v*_*lac*_/*µ* ratio) can improve lactate production, but excessive overexpression of this pathway may sacrifice biomass growth and impair overall lactate productivity. To resolve this problem, it is desirable to induce the lactate synthesis pathway at late biomass growth phase.

Deleting carbon storage in cyanobacteria is one strategy to redirect carbon flux to product synthesis [30]. However, the integrated GSM shows that such a strategy may not offer significant improvements in final lactate productivity in PBRs (Fig. 6a, d, e). This finding is consistent with two recent reports: (1) Glycogen knockout did not enhance lactate productivity under nutrient-sufficient growth conditions [30]. (2) Removal of glycogen in an isobutanol-producing cyanobacterium yielded no benefit in production titer and rate [31]. Possibly, glycogen serves as the carbon and energy reservoir to store the energy and carbon excess flow in the light zone, and this carbon and energy reservoir can maintain redox homeostasis under stressed growth conditions or in darkness [32]. Therefore, deleting glycogen or other carbon storage may impair cyanobacterial survival as well as its resistance to environmental stresses and contaminations.

## Discussion

In this study, a genome-scale FBA model was integrated with information on kinetics, light distribution, and cell movement. Using the integrated GSM, one can simultaneously learn both intracellular information (e.g., flux distributions as functions of time) and extracellular information (e.g., growth curve and nutrient changes in the medium) simultaneously.

In the extracellular domain, the integrated GSM can describe changes in nutrient concentrations, biomass accumulation, and local light intensities. As demonstrated by Fig. 3, cyanobacteria are intrinsically capable of reaching high biomass concentration in PBRs, however, their performance is usually limited by low light availability and low mass transfer rate. To reduce cell self-shading, high surface-to-volume ratio PBRs equipped with thin panel or hollow fibers have been employed [23]. To improve the mass transfer rate, enhancing aeration rates has proved efficient [19]. Better mixing conditions not only lead to better gas transfer rates, but also help maintain more homogeneous conditions for both cells and nutrients.

In the intracellular domain, we observed continuously changing fluxes in the cyanobacterial metabolic network, which were mostly affected by reduced energy and carbon inputs. One interesting finding is the OPP pathway activation as cell cultures get denser. It is a traditional point of view that the Calvin cycle and the OPP pathway are separate systems because the two pathways are reciprocally regulated [33]. In this study, the integrated GSM describes that these two pathways could be employed by two different subpopulations in PBRs at the same time. This simulation explains that a measureable flux through the OPP pathway could be observed in both wild type and engineered cyanobacterial strains via ^13^C-based flux analysis [24, 25]. In addition, the elementary modes analysis shows that the Calvin cycle and the OPP pathway may function in complementary ways in photoautotrophs, since an active OPP pathway ensures a maintainable flux to triose phosphate synthesis from carbohydrate degradation in low light or darkness [33]. Furthermore, we noticed that an active OPP pathway was always present in the d-lactate producing strain, and it became stronger with increased d-lactate production (Additional file 2: Fig. S7). This indicates that the enhanced OPP activity benefits bio-production by providing more reducing power. In summary, the plasticity of the OPP pathway endows cells with high vitality and energy flexibility [34, 35].

The traditional FBA model usually describes the optimal growth condition, and thus it may not be suitable for suboptimal and heterogeneous cultivation conditions. Our model, on the other hand, is integrated with growth kinetics and a heterogeneous light distribution in PBRs. Hence, the model can not only predict the production yield, titer and rate, but also offer insights into how cells adjust their internal metabolisms to survive under different growth conditions and genetic manipulations. Moreover, the integrated GSM may give more accurate predictions of mutant physiology than GSM alone in bioreactor conditions. For example, the integrated GSM correctly indicates that glycogen knockout may not be an effective strategy to improve PBR lactate production. Lastly, the integrated GSM can reveal real-time variations/dynamics in metabolisms of different subpopulation cells, and thus improve understandings of cellular responses to large-size PBRs.

Nevertheless, our model still has limitations. For example, previous studies have shown that glycogen synthesis could be connected with unknown regulations affecting cyanobacterial viability under stress conditions [32, 36]. However, the integrated GSM may not give the same prediction without further constraints from knowledge of genetic regulations. Additionally, it has been demonstrated that cyanobacteria have circadian behaviors (i.e., their metabolism exhibits day and night rhythms) [37], while our model does not include this property. Finally, some inhibition factors may also influence cyanobacterial growth (e.g., effects of crowding), which are not included in the model. In the future, this model platform should be further improved via additional multi-scale modeling approaches.

## Conclusion

This study demonstrates a genome-scale FBA model integrated with kinetics, cell movements, and a light distribution function. With constraints obtained from bioprocess variables, the integrated GSM can not only simulate the dynamic metabolisms in sub-population cells but also predict PBR overall productivity under light and CO_2_ conditions. The integration of GSMs with PBR modeling can facilitate the development of new cyanobacterial strains for industrial settings.

## Methods

### Cell cultivation

*Synechocystis* PCC 6803 was cultivated in a modified BG-11 medium [26] at 30 °C and 180 rpm. We first tested the cyanobacterial growth in different culture volumes. In brief, 50, 100, and 150 mL of cell suspensions were cultivated in 250 mL shake flasks under continuous illumination of ~50 *µ*E/m^2^/s. We also tested the cyanobacterial growth under different light conditions. Specifically, 15 mL of cultures were grown in 150 mL shake flasks under different light intensities (from ~15 to ~35 *µ*E/m^2^/s). OD_730_ was used to measure biomass density, and the relationship between the biomass concentration and OD_730_ was 0.45 × OD_730_ = Biomass (g/L) [26]. We made duplicate cultures of each condition (n = 2).

## ^13^C-Labelling experiment

^13^C-labeling experiments were performed to determine histidine labeling as evidence of OPP pathway activity under different light conditions. We grew photomixotrophic cultures in BG-11 medium supplied with 2.5 g/L [1-^13^C] glucose and 4 g/L NaH^13^CO_3_ (tracers were purchased from Sigma-Aldrich, Saint Louis, USA). The TBDMS (*N*-tert-butyldimethylsilyl-*N*-methyltrifluoroacetamide) method [26] was used to analyze the labeling patterns of proteinogenic histidine. In brief, cells were harvested by centrifugation, and cell pellets were hydrolyzed in 6 mol/L HCl solution at 100 °C for 24 h. The amino acid solution was air-dried and then derivatized by TBDMS (Sigma-Aldrich, USA) at 70 °C for 1 h. A gas chromatograph (GC) (Hewlett-Packard model 7890A; Agilent Technologies, CA) equipped with a DB5-MS column (J&W Scientific, Folsom, CA) and a mass spectrometer (model 5975C; Agilent Technologies, CA) were used for analyzing amino acid labeling profiles. The GC–MS fragment [M-57]^+^ contains the complete amino acid backbone, and MS data M + 0, M + 1, and M + 2 represent isotopomers with zero, one, and two ^13^C atoms, respectively.

### Flux balance analysis model

The FBA model was modified from the cyanobacterial model iJN878 [15], which has 843 reactions, including photosynthesis and the central carbon metabolism. A complete list of reactions is provided in Additional file 1. The iJN878 model contains a recently discovered γ-aminobutyrate shunt [38] which converts 2-oxoglutarate to succinate in *Synechocystis* 6803. In our model, two new reactions were added, namely ‘glycogen storage → glycogen [c]’ and ‘d-lactate [c] → d-lactate [external]’, which were respectively used to simulate glycogen storage/consumption and d-lactate production by an engineered cyanobacterial strain [29]. The mathematical description of our FBA model is as follows:

1 $$\left[ \begin{array}{l} {\text{maximize }}\mu \hfill \\ {\text{subject to }}S \cdot v = 0 \hfill \\ \quad \quad \quad \quad lb \le v \le ub \hfill \\ \quad \quad \quad \quad v_{{CO_{2} }} \le f_{1} \left( {K_{{La}} ,[{\text{CO}}_{2} ],Km} \right) \hfill \\ \quad \quad \quad \quad v_{{photon}} \le f_{2} \left( {l,X,v_{{photon,0}} } \right) \hfill \\ \end{array} \right],$$

where *µ* represents the specific growth rate, *S* is the stoichiometric matrix, *v* represents a vector of flux distribution, and *lb* and *ub* represent vectors of the lower and upper boundaries, respectively. Further, *f*_*1*_ is a function of the mass transfer coefficient *K*_*La*_, dissolved CO_2_ concentration [CO_2_], and half-saturation constant for dissolved CO_2_, *Km*; *f*_*2*_ is a function of the cell’s local position *l*, biomass concentration *X*, and photon influx on the PBR surface *v*_*photon,0*_. The linear optimization problem was solved by the MATLAB^®^ (2012b) built-in function ‘linprog’ using the ‘simplex’ algorithm. To estimate the flux distribution in engineered cyanobacterial strains, we used the MOMA (minimization of metabolic adjustment) algorithm [39], which was solved by the MATLAB built-in function ‘quadprog’ using the ‘interior-point-convex’ algorithm:

2 $$\left[ \begin{array}{l} \text{minimize} \, \frac{1}{2}v^{T} Hv - f_{opt}^{T} v \hfill \\ {\text{subject to }}S \cdot v = 0 \hfill \\ \quad \quad \quad \quad lb \le v \le ub \hfill \\ \quad \quad \quad \quad v_{{CO_{2} }} \le f_{1} \left( {K_{La} , [ {\text{CO}}_{2} ],Km} \right) \hfill \\ \quad \quad \quad \quad v_{photon} \le f_{2} \left( {l,X,I_{0} } \right) \hfill \\ \end{array} \right],$$

where *H* is a unit matrix, and *f*_*opt*_ is the optimal flux distribution of wild-type cyanobacteria. The remaining notations have the same meanings as above.

Moreover, we considered three growth states for cyanobacteria in the FBA model: (1) the ‘light condition’: an autotrophic sub-population in the light zone, (2) the ‘dark condition’: a heterotrophic sub-population in the dark zone, where the photon influx is below 0.4 mmol/g/h (under which the cyanobacterial growth rate is lower than the heterotrophic growth rate in darkness) and glycogen is consumed at a rate of 0.01 mmol/g/h [40] to maintain minimal growth, and (3) the ‘resting condition’: a glycogen-depleted sub-population with no active fluxes in the dark zone. To improve the calculation efficiency, we built a database containing all the flux distributions in response to different photon influxes (Additional file 1). By having such a database, we could directly use pre-calculated fluxome from the database according to culture conditions in PBRs. Thereby, we did not need to redo flux calculations at each time interval during new simulations.

### Simulation of cyanobacterial growth via integrating FBA, kinetics, and cell movements

Figure 1 shows our modeling algorithm. To simulate biomass growth as a function of time, we divided the entire time period into finite intervals of 0.002 h (Additional file 2: Fig. S1 shows that further decreasing the interval period did not change the simulation results). In each time interval, a simplified sinusoid equation [41] was used to estimate the cell location in a well-mixed PBR:

3 $$l = \frac{r}{2} - \frac{r}{2}\cos (\frac{2\pi }{{f_{r} }}t),$$

where *l* is the shortest distance between the PBR surface and the cell local position, in mm; *r* is the radius or thickness of the PBR, in mm; *f*_*r*_ represents the cyanobacteria circulation frequency, in h; and *t* is time, in h. Because cell circulation frequencies in PBRs vary from cell to cell, stochastic effects are induced on a single cell’s metabolism. In fact, the random movements of cells in PBRs have been measured and simulated, and, in the present study, are described by a probability distribution function [42]. In our model platform, we distinguished cell populations with different circulation times (Additional file 2: Fig. S2-3). Thus, the whole culture was considered to be comprised of twelve sub-populations instead of a plethora of cyanobacterial cells. Based on cell locations and the biomass concentrations, we calculated the local light intensity [43]:

4 $$\frac{{v_{photon} }}{{v_{photon,0} }} = \left( {\frac{1}{{(0.0216 \cdot l + 1)^{1.54} (0.130 \cdot X \cdot + 1)^{1.18} }}} \right),$$

where *v*_*photon*_ and *v*_*photon,0*_ represent the local and surface photon influxes, respectively, in mmol/g/h; and X is biomass concentration, in g/L.

The CO_2_ uptake flux was described by a Michaelis–Menten equation:

5 $$v_{{{\text{CO}}_{2} }} = v_{{{\text{CO}}_{2} \,,\hbox{max} }} \frac{{[{\text{CO}}_{2} ]}}{{K_{m} + [{\text{CO}}_{2} ]}},$$

where *v*_CO2*,max*_ is the maximum uptake rate of dissolved CO_2_/HCO_3_^−^, *K*_*m*_ is the half-saturation constant, and [CO_2_] represents dissolved CO_2_ concentration. This study assumed that pH was constant at 8.0, and that the dissolved CO_2_ and cell culture were homogeneous in PBRs. Because we assumed that cell metabolism was pseudo-steady in each interval [44], the FBA model could use linear optimization to profile the intracellular fluxes constrained by light and carbon input fluxes (Eqs. 4 and 5). The FBA model then predicted the growth rates, glycogen synthesis rates, and CO_2_ uptake rates of cell populations with different circulation times in PBRs. Those values were averaged based on the probability distribution function (Additional file 2: Fig. S2a):

6 $$\begin{aligned} \mu _{{app}} & = \sum\limits_{{i = 1}}^{n} {P_{i} \mu _{i} } \\ v_{{{\text{CO}}_{2} ,app}} & = \sum\limits_{{i = 1}}^{n} {P_{i} v_{{{\text{CO}}_{2} ,i}} } \\ v_{{glycogen,app}} & = \sum\limits_{{i = 1}}^{n} {P_{i} v_{{glycogen,i}} } , \\ \end{aligned}$$

where *P*_*i*_ is the fraction of *i*th cell population (Additional file 2: Fig. S2a), *µ*_*app*_ is the apparent specific growth rate in PBRs, *v*_CO2*,app*_ is the apparent CO_2_ uptake rate, and *v*_*glycogen,app*_ is the overall glycogen production rate.

For the kinetic model, we used ordinary differential equations (ODEs) to describe changes in biomass production, glycogen accumulation, dissolved CO_2_/HCO_3_^−^ concentrations, and so forth.

7 $$\begin{aligned} \frac{dX}{dt} &= \mu_{app} \cdot X - K_{d} \cdot X \hfill \\ \frac{d[glycogen]}{dt} &= v_{glycogen,app} \cdot X - \beta \cdot K_{d} \cdot X \hfill \\ \frac{{d[{\text{CO}}_{2} ]}}{dt} &= K_{La} \cdot ([{\text{CO}}_{2} ]^{*} - [{\text{CO}}_{2} ]) + v_{{{\text{CO}}_{2} ,app}} \cdot X, \hfill \\ \end{aligned}$$

The ODEs were resolved in their numerical discrete form (Euler-like integration scheme):

8 $$\begin{aligned} X_{i + 1} = X_{i} + \mu_{app} \cdot X_{i} \cdot \Delta t - K_{d} \cdot X_{i} \cdot \Delta t \hfill \\ [glycogen]_{i + 1} = [glycogen]_{i} + v_{glycogen,app} \cdot X_{i} \cdot \Delta t - \beta \cdot K_{d} \cdot X_{i} \cdot \Delta t \hfill \\ [{\text{CO}}_{2,i + 1} ] = [{\text{CO}}_{2,i} ] + K_{La} \cdot ([{\text{CO}}_{2} ]^{*} - [{\text{CO}}_{2,i} ]) \cdot \Delta t + v_{{{\text{CO}}_{2} ,app}} \cdot X_{i} \cdot \Delta t \hfill \\ \end{aligned}$$

where *i* and *i* + 1 represent the current and next intervals, respectively; *Δt* is the time interval (0.002 h); *K*_*d*_ is the death rate, in h^−1^; *β* is the glycogen composition in the biomass, in mmol/g; [*glycogen*] is the overall glycogen concentration in the PBR, in mmol/L; *K*_*La*_ is the mass transfer rate of CO_2_, in h^−1^; [CO_2_] represents the dissolved CO_2_ and HCO_3_^−^ concentrations, in mmol/L; [CO_2_]^*^ is the combined concentrations of dissolved CO_2_ and HCO_3_^−^ in equilibrium with atmospheric CO_2_ (0.039 %, v/v), in mmol/L; and *µ*_*app*_, *v*_*glycogen,app*_, and *v*_CO2*,app*_ are fluxes determined previously. The updated values of the biomass concentration, dissolved CO_2_ concentration, etc., were then used to constrain the FBA model in the next interval. The kinetic parameters are given in Table 1. For shake flask conditions, *K*_*La*_ was determined by the following equation: [45]

**Table 1:** List of parameters used to simulate the growth and metabolic fluxes of cyanobacteria growing in a cylindrical PBR

Parameters	Significance	Value (range)	Unit	References/notes
*K*_*La*_	Mass transfer rate of CO_2_	10^a^ (3–15)	h^−1^	[19]
*K*_*d*_	Death rate	0.0079	h^−1^	[46]
Radius	Radius of PBR	60	mm	Similar to the reactor used in Reference [42]
*I*_*0*_	Surface light intensity	50	mmol/g/h	Equivalent to ~50 *µ*E/m^2^/s^b^
*Km*	Half-saturation constant of CO_2_ uptake rate	8	*µ*mol/L	[47]
*pH*	Medium pH	8.0	unitless	BG-11 medium
[*X*]_0_	Initial biomass concentration	0.1	g/L	Equivalent to an OD_730_ of ~0.2
[CO_2_]_0_	Initial concentration of dissolved CO_2_ and HCO_3_^−^ (in equilibrium with air)	0.53	mmol/L	Estimated^c^

^a^The value is used in Figs. 2, 3 and 5; ^b^a photosynthesis efficiency of 1.5 % is assumed in Figs. 2, 3 and 5; and ^c^calculation is based on experimental conditions (Additional file 2)

9 $${\it{\text{K}}}_{\text{La}} { \,= 0} . 0 3 2 \times {\text{N}}\left( {\frac{\text{V}}{\text{L}}} \right)^{ 0. 8 4 5} ,$$

where *N* is the rotation speed, in rpm; *V* is the shake flask volume, in mL; and *L* is the culture volume, in mL. MATLAB code of the integrated GSM is provided in Additional file 3 and 4.

## Additional files

**Additional file 1.** Flux distribution database.

**Additional file 2.** Supplementary figures and tables.

**Additional file 3.** MATLAB file for simulating cyanobacterial performance in PBRs.

**Additional file 4.** Matrix of flux distribution database.
