# Supplementary material for: Simulating cyanobacterial phenotypes by integrating flux balance analysis, kinetics, and a light distribution function
Source: Microb Cell Fact. 2015 Dec 24;14:206. doi: 10.1186/s12934-015-0396-0 (PMC5574461; doi:10.1186/s12934-015-0396-0)
Supplement: Supplementary file 2 — Additional file 2. Supplementary figures and tables. [file 12934_2015_396_MOESM2_ESM.docx]

**Simulating cyanobacterial phenotypes by integrating flux balance analysis, kinetics, and a light distribution function**

**Additional file 2**

Lian He^1^, Stephen G. Wu^1^, Ni Wan^2^_,_ Adrienne C. Reding^3^ and Yinjie Tang^1*^

1. Department of Energy, Environmental and Chemical Engineering, Washington University, St. Louis, MO 63130, USA.

2. Department of Mechanical Engineering and Materials Science, Washington University, St. Louis, MO 63130, USA.

3. Department of Biochemistry and Molecular Biology, College of Wooster, Wooster, OH 44691, USA.

Running title: dynamic FBA model for heterogenous cyanobacterial cultrues

^*^ Corresponding Author: E-mail: [yinjie.tang@seas.wustl.edu](mailto:yinjie.tang@seas.wustl.edu). Telephone: 314-935-3441.

Contents

[Table S1 Parameters used for simulating cyanobacterial growth in shake flasks. 3](#_Toc437254286)

[Figure S1 The influence of time intervals on cyanobacterial growth. 4](#_Toc437254287)

[Figure S2. Probability distribution of cell circulation time in photobioreactors. 5](#_Toc437254288)

[Figure S3 Cyanobacteria growth with different probability distributions. 6](#_Toc437254289)

[Figure S4 Specific growth rates and fluxes through the oxidative pentose phosphate pathway (OPP) under heterotrophic growth conditions in darkness 7](#_Toc437254290)

[Figure S5 Simplified geometry of shake flask. 8](#_Toc437254291)

[Figure S6 Determining the photosynthesis efficiency of cyanobacteria growing in 250 mL shake flasks. 9](#_Toc437254292)

[Figure S7 Comparison of flux distributions (unit: mmol/g/h) in central metabolism between wild-type, glycogen knockout, and D-lactate producing cyanobacterial strains. 10](#_Toc437254293)

[Additional information for the integrated FBA model 12](#_Toc437254294)

[References 13](#_Toc437254295)

# Table S1 Parameters used for simulating cyanobacterial growth in shake flasks.

The remaining parameters used in the model are the same as shown in Table 1 in the article.

| **Parameters** | **Significance** | **Value**  **(Range)** | **Unit** | **Reference/Notes** |
| --- | --- | --- | --- | --- |
| *K_La_* | Mass transfer rate of CO_2_ | 8-55 | h^-1^ | Calculated based on reference [[1](#_ENREF_1)] |
| *K_d_* | Death Rate | 0 | h^-1^ | n/a |
| Thickness | Thickness of the culture | ~5，~15, ~30, and ~45 | mm | Measured for,15 mL, 50 mL,100 mL, and 150 mL cell cultures, respectively, in 250 mL shake flasks |
| *X_0_* | Initial biomass concentration | 0.01 | g/L | Measured from the experiment |

# **Figure S1 The influence of time intervals on cyanobacterial growth.**

We tested different time intervals in the integrated FBA model, and then we plotted the biomass growth as a function of time. The results show that choosing a time interval between 0.001-0.005 h is good for our model.

# Figure S2. Probability distribution of cell circulation time in photobioreactors.

The distribution is based on the reference [[2](#_ENREF_2)], in which the circulation times follow the normal distribution with the mean and standard deviation being 10 s and 1.3 s, respectively. Instead of using a continuous probability function, we used a discrete probability mass function shown below (Figure a). Figure b and c show the oscillations of specific growth rate and cell distance to the PBR surface in 10 minutes, respectively.

# **Figure S3 Cyanobacteria growth with different probability distributions.**

We predicted cyanobacterial growth in a 60mm-radius cylindrical PBR with a constant external photon influx of 50 *µ*E/m^2^/s and a *K_La_* value of 10 h^-1^. Different circulation times are tested. The results show that the circulation time does not affect the total PBR biomass productions. The growth curves shown below overlap each other.

# **Figure S4 Specific growth rates and fluxes through the oxidative pentose phosphate pathway (OPP) under heterotrophic growth conditions in darkness**

# **Figure S5 Simplified geometry of shake flask.**


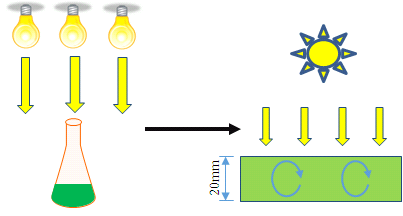


Unlike previous simulations, we simplified the geometry of the shake flasks into a two-dimensional rectangle, and made the local light intensity dependent on the vertical distance from a cell to the light source.

# Figure S6 Determining the photosynthesis efficiency of cyanobacteria growing in 250 mL shake flasks.

**Best fit is achieved at 2.7%.**

# Figure S7 Comparison of flux distributions (unit: mmol/g/h) in central metabolism between wild-type, glycogen knockout, and D-lactate producing cyanobacterial strains.


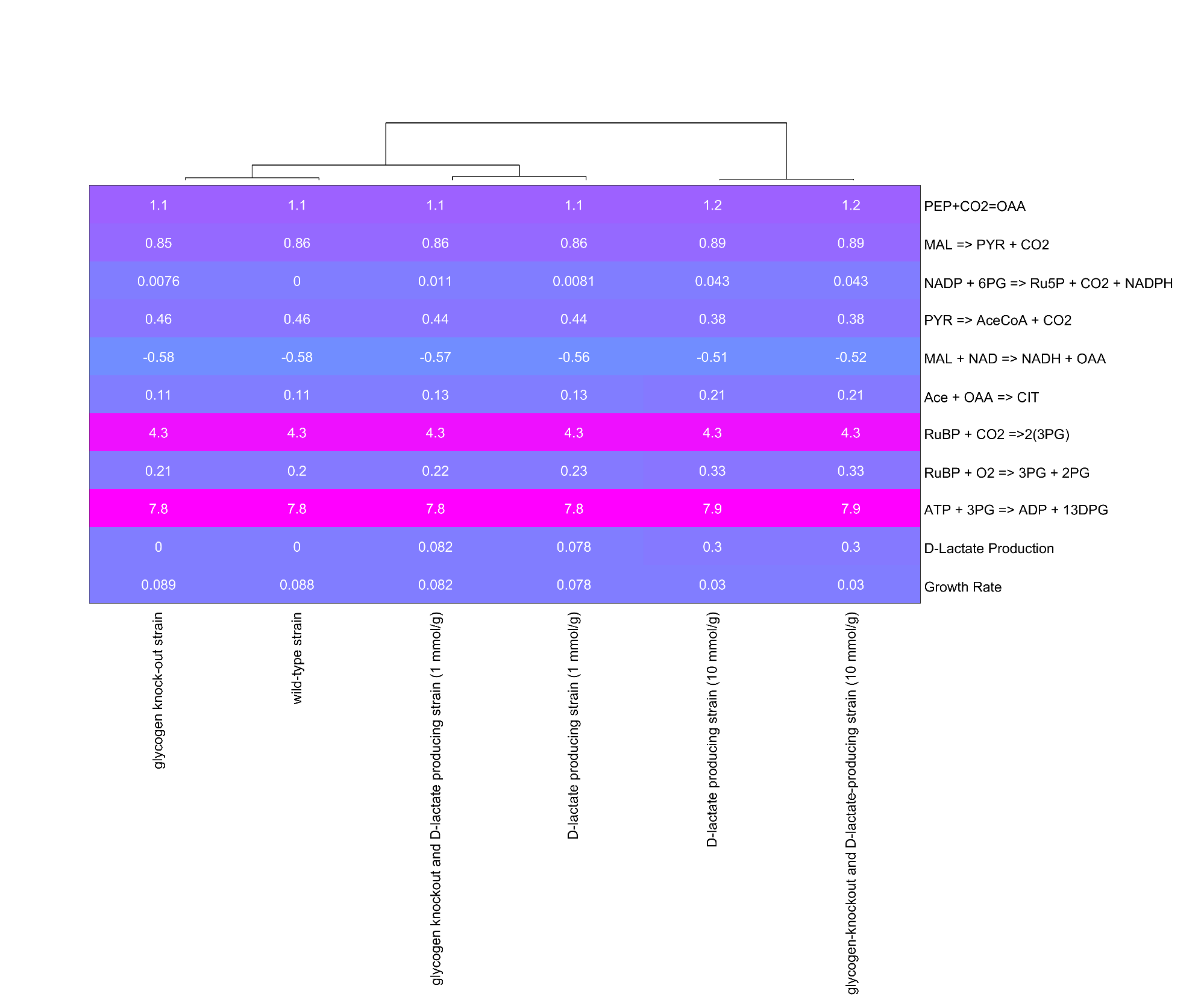


Legends: A, wild-type strain; B, glycogen-knockout strain; C, D-lactate producing strain (1 mmol/g); D, glycogen knockout and D-lactate producing strain (1 mmol/g); E, D-lactate producing strain (10 mmol/g); and F, glycogen-knockout and D-lactate-producing strain (10 mmol/g). The model assumes lactate production to be 1mmol/g biomass or 10 mmol/g biomass. Unlike the wild-type strain, lactate-producing cyanobacteria with a *v_lac_*/*µ* of 1 mmol/g appears to have an active OPP flux (0.01 mmol/g/h). As *v_lac_*/*µ* is increased to 10 mmol/g, the OPP flux becomes more active (0.04 mmol/g/h). An active OPP flux can be beneficial by providing more NADPH for lactate synthesis.

# Additional information for the integrated FBA model

**(1) The mass transfer coefficients of CO_2_ and O_2_** [[3](#_ENREF_3)].

,

where *K_La_CO2_* and *K_La_O2_* are the mass transfer rates of CO_2_ and O_2_, respectively; and *D_CO2_* and *D_O2_* are the diffusion coefficients of CO_2_ and O_2_, respectively.

**(2) Estimation of dissolved CO_2_ in equilibrium with ambient air. The parameters are based on the textbook Water Chemistry [**[**4**](#_ENREF_4)**].**

$\frac{[H^{+}][HCO_{3}^{-}]}{[H_{2}CO_{3}]}=10^{-6.4}$ $\frac{[H^{+}][CO_{3}^{2-}]}{[HCO_{3}^{-}]}=10^{-10.3}$

In this study, we assumed that pH was constant as 8.0. Thus the total carbon source available in the medium would be 0.53mM. Following is a table showing [CO_2_/H_2_CO_3_] + [HCO_3_^-^] at different pH.

List of bioavailable CO_2_ concentrations at different pH in medium

| CO_2_ content in the gas phase | pH of medium | [CO_2_/H_2_CO_3_]+[HCO_3_^-^]  (in equilibrium with air) |
| --- | --- | --- |
| 0.039% | 8.0 | 0.53mM |
| 0.039% | 7.5 | 0.18mM |
| 0.039% | 7.0 | 0.065mM |
| 3% | 8.0 | 42mM |

Note: (1) *p_CO2_* is the partial pressure of CO_2_ in the air; (2) *H_CO2_* is the Henry constant of CO_2_; and (3): ‘mM’ means ‘mmol/L’.

# References

1. Nikakhtari H, Hill GA: **Modelling oxygen transfer and aerobic growth in shake flasks and well-mixed bioreactors.** *Can J Chem Eng* 2005, **83:**493-499.

2. Luo H-P, Al-Dahhan MH: **Verification and validation of CFD simulations for local flow dynamics in a draft tube airlift bioreactor.** *Chem Eng Sci* 2011, **66:**907-923.

3. Royce PN, Thornhill NF: **Estimation of dissolved carbon dioxide concentrations in aerobic fermentations.** *AIChE J* 1991, **37:**1680-1686.

4. Benjamin MM: *Water chemistry.* McGraw-Hill New York; 2002.
