# Supplementary material for: Simulating cyanobacterial phenotypes by integrating flux balance analysis, kinetics, and a light distribution function
Source: Microb Cell Fact. 2015 Dec 24;14:206. doi: 10.1186/s12934-015-0396-0 (PMC5574461; doi:10.1186/s12934-015-0396-0)
Supplement: Supplementary file 4 — Additional file 4. Matrix of flux distribution database. [file 12934_2015_396_MOESM4_ESM.docx]

clc

clear

%% Initial values and parameters

load AllFluxRecord

Glycogenratio=0.21031*1;

% Initial Conditions_highglycogen

Mu_ave=0.0884;% h-1

X=0.1;%0.1;%g/L %

Glycogen=X*Glycogenratio;%g/L

O2=0.26;%mmol/L

CO2=0.53;%mmol/L 42;%

NO3=18;%mmol/L

PO4=0.3;%mmol/L

I0=50;%mmol/g/h (1.5 % photosynthesis efficiency)

radius=60;%mmm

% Equilibrium Condition

CO2_constant=0.53;%mmol/L42;%

O2_constant=0.26;%mmol/L

% Parameters

timemax=21*24;%h

step1=0.002;%h every 7.2s

distributionmax=12;% divide each cycle into 20 invervals

step2=1;%

Kla1=10;%h-1 for CO2 10 (bioreactor) 62*0.89(15ml) 22*0.89(50ml) 12*0.89(100ml) 9*0.89(150ml)

Kla2=Kla1/0.89;%h-1 for O2

Kd=0.19/24;%h-1;%0;%

% number of time and distribution intervals

numoftime=round(timemax/step1);

numofdistribution=distributionmax/step2;

% Matrices and vectors for recording values of parameters over time

I_record=zeros(numofdistribution,numoftime);

MU_record=zeros(numofdistribution,numoftime);

vg_record=zeros(numofdistribution,numoftime);

vco2_record=zeros(numofdistribution,numoftime);

vo2_record=zeros(numofdistribution,numoftime);

vno3_record=zeros(numofdistribution,numoftime);

vpo4_record=zeros(numofdistribution,numoftime);

location=zeros(numofdistribution,numoftime);

% Record flux distributions

R136_record=zeros(1,numoftime);%Ace+OAA=CIT

R298_record=zeros(1,numoftime);%NADP+6PG=Ru5P+CO2+NADPH

R359_record=zeros(1,numoftime);%MAL+NAD=NADH+OAA

R464_record=zeros(1,numoftime);%ATP+3PG=ADP+13DPG

R502_record=zeros(1,numoftime);%PEP+CO2=OAA

R549_record=zeros(1,numoftime);%RuBP+O2=>3PG+2PG

R554_record=zeros(1,numoftime);%RuBP+CO2=>2(3PG)

R585_record=zeros(1,numoftime);%Sucsal=>Suc (The last step of GABA shunt)

X_record=zeros(1,numoftime);

Glycogen_record=zeros(1,numoftime);

CO2_record=zeros(1,numoftime);

O2_record=zeros(1,numoftime);

NO3_record=zeros(1,numoftime);

PO4_record=zeros(1,numoftime);

Iave_record=zeros(1,numoftime);

Muave_record=zeros(1,numoftime);

I0_record=zeros(1,numoftime);

Population=zeros(3,numoftime);% 1 represents the autotrophic cultures; 2 represents the heterotrophic culture; 3 represents the resting cell

%circulation frequency and probability distribution

pdf=[5,0.011;

6,0.024;

7,0.057;

8,0.105;

9,0.157;

10,0.187;

11,0.178;

12,0.136;

13,0.083;

14,0.040;

15,0.016;

16,0.006;];

Probability=pdf(:,2);

frequency=pdf(:,1)+0;

mumax=0.08838454;%

VCO2_critical=3.7*CO2/(CO2+8/1000);%8 for low CO2 130 for high CO2

dCO2=round(VCO2_critical/0.0068398);

VNO3_critical=NO3/X/step1;

dNO3=round(VNO3_critical/0.0014726);

VPO4_critical=0.060955*PO4/(PO4+1/1000);

dPO4=round(VPO4_critical/1.1268097e-04);

Vg_critical=Glycogen/X/step1;

%% integration of kinetic model, fluid dynamic model, with FBA results

for time=step1:step1:timemax %time

% Create vectors for record

VMu=zeros(numofdistribution,1);

Vglycogen=zeros(numofdistribution,1);

VCO2=zeros(numofdistribution,1);

VO2=zeros(numofdistribution,1);

VNO3=zeros(numofdistribution,1);

VPO4=zeros(numofdistribution,1);

I_local=zeros(numofdistribution,1);

% Record flux distributions

R136=zeros(numofdistribution,1);% Ace+OAA=CIT

R298=zeros(numofdistribution,1);% NADP+6PG=Ru5P+CO2+NADPH

R359=zeros(numofdistribution,1);% MAL+NAD=NADH+OAA

R464=zeros(numofdistribution,1);% ATP+3PG=ADP+13DPG

R502=zeros(numofdistribution,1);% PEP+CO2=OAA

R549=zeros(numofdistribution,1);% RuBP+O2=>3PG+2PG

R554=zeros(numofdistribution,1);% RuBP+CO2=>2(3PG)

R585=zeros(numofdistribution,1);% GABA Shunt

for circulation=step2:step2:distributionmax

% Cyanobacteria positions

la=radius/2-radius/2*(cos((22608/frequency(circulation,1)*(time))));

if la>radius;la=radius;end

if la<0;la=0;end

location(round(circulation/step2),round(time/step1))=la;

% Boundary constraint for light intensity

I=I0/((0.0216*la+1)^(1.54))/((0.130*X*la+1)^(1.18));

I_local(round(circulation/step2))=I;

light_intensity=round(I*10);

if I>=55

light_intensity=550;

end

limit=round(min(light_intensity,dCO2));

%limit=round(min(light_intensity,dCO2,dNO3,dPO4));

if limit>4 && CO2>0 % light zone 13(glycogen high) 4 (normal)

xbest=AllFluxRecord(2:end,limit);

VMu(round(circulation/step2))=xbest(808);

Vglycogen(round(circulation/step2))=xbest(808)*Glycogenratio-xbest(864);

VCO2(round(circulation/step2))=xbest(766);

VO2(round(circulation/step2))=xbest(761);

VNO3(round(circulation/step2))=xbest(776);

VPO4(round(circulation/step2))=xbest(781);

R136(round(circulation/step2))=xbest(136);%Ace+OAA=CIT

R298(round(circulation/step2))=xbest(298);%NADP+6PG=Ru5P+CO2+NADPH

R359(round(circulation/step2))=xbest(359);%MAL+NAD=NADH+OAA

R464(round(circulation/step2))=xbest(464);%ATP+3PG=ADP+13DPG

R502(round(circulation/step2))=xbest(502);%PEP+CO2=OAA

R549(round(circulation/step2))=xbest(549);%RuBP+O2=>3PG+2PG

R554(round(circulation/step2))=xbest(554);%RuBP+CO2=>2(3PG)

R585(round(circulation/step2))=xbest(585);% GABA shunt

Population(1,round(time/step1))=Population(1,round(time/step1))+Probability(circulation);

else if light_intensity<=4 && Vg_critical>=0.01 && CO2>0 % dark zone

xbest=AllFluxRecord(2:end,end);

VMu(round(circulation/step2))=xbest(810);

Vglycogen(round(circulation/step2))=xbest(808)*Glycogenratio-xbest(864);

VCO2(round(circulation/step2))=xbest(766);

VO2(round(circulation/step2))=xbest(761);

VNO3(round(circulation/step2))=xbest(776);

VPO4(round(circulation/step2))=xbest(781);

R136(round(circulation/step2))=xbest(136);%Ace+OAA=CIT

R298(round(circulation/step2))=xbest(298);%NADP+6PG=Ru5P+CO2+NADPH

R359(round(circulation/step2))=xbest(359);%MAL+NAD=NADH+OAA

R464(round(circulation/step2))=xbest(464);%ATP+3PG=ADP+13DPG

R502(round(circulation/step2))=xbest(502);%PEP+CO2=OAA

R549(round(circulation/step2))=xbest(549);%RuBP+O2=>3PG+2PG

R554(round(circulation/step2))=xbest(554);%RuBP+CO2=>2(3PG)

R585(round(circulation/step2))=xbest(585);% GABA shunt

Population(2,round(time/step1))=Population(2,round(time/step1))+Probability(circulation);

else

VMu(round(circulation/step2))=0;

Vglycogen(round(circulation/step2))=0;

VCO2(round(circulation/step2))=0;

VO2(round(circulation/step2))=0;

VNO3(round(circulation/step2))=0;

VPO4(round(circulation/step2))=0;

R136(round(circulation/step2))=0;%Ace+OAA=CIT

R298(round(circulation/step2))=0;%NADP+6PG=Ru5P+CO2+NADPH

R359(round(circulation/step2))=0;%MAL+NAD=NADH+OAA

R464(round(circulation/step2))=0;%ATP+3PG=ADP+13DPG

R502(round(circulation/step2))=0;%PEP+CO2=OAA

R549(round(circulation/step2))=0;%RuBP+O2=>3PG+2PG

R554(round(circulation/step2))=0;%RuBP+CO2=>2(3PG)

R585(round(circulation/step2))=0;%GABA shunt

Population(3,round(time/step1))=Population(3,round(time/step1))+Probability(circulation);

end

end

end

% Record all the results

MU_record(:,round(time/step1))=VMu;

vg_record(:,round(time/step1))=Vglycogen;

vco2_record(:,round(time/step1))=VCO2;

vo2_record(:,round(time/step1))=VO2;

vno3_record(:,round(time/step1))=VNO3;

vpo4_record(:,round(time/step1))=VPO4;

I_record(:,round(time/step1))=I_local;

I0_record(:,round(time/step1))=I0;

% Average value of ouput fluxes

Mu_ave=sum(VMu.*Probability);

Vg_ave=sum(Vglycogen.*Probability);

VCO2_ave=sum(VCO2.*Probability);

VO2_ave=sum(VO2.*Probability);

VNO3_ave=sum(VNO3.*Probability);

VPO4_ave=sum(VPO4.*Probability);

Iave=sum(I_local.*Probability);

R136_record(round(time/step1))=sum(R136.*Probability);%Ace+OAA=CIT

R298_record(round(time/step1))=sum(R298.*Probability);%NADP+6PG=Ru5P+CO2+NADPH

R359_record(round(time/step1))=sum(R359.*Probability);%MAL+NAD=NADH+OAA

R464_record(round(time/step1))=sum(R464.*Probability);%ATP+3PG=ADP+13DPG

R502_record(round(time/step1))=sum(R502.*Probability);%PEP+CO2=OAA

R549_record(round(time/step1))=sum(R549.*Probability);%RuBP+O2=>3PG+2PG

R554_record(round(time/step1))=sum(R554.*Probability);%RuBP+CO2=>2(3PG)

R585_record(round(time/step1))=sum(R585.*Probability);%GABA shunt

% Calculate the current biomass and glycogen concentration

X_new=X+Mu_ave*X*step1-Kd*X*step1;

Glycogen_new=Glycogen+Vg_ave*X*step1-Kd*X*step1*Glycogenratio;%

O2_new=O2+Kla2*(O2_constant-O2)*step1+VO2_ave*X*step1;

CO2_new=CO2+Kla1*(CO2_constant-CO2)*step1+VCO2_ave*X*step1;

NO3_new=NO3+VNO3_ave*X*step1;

PO4_new=PO4+VPO4_ave*X*step1;

% record the new values

X_record(round(time/step1))=X_new;

Glycogen_record(round(time/step1))=Glycogen_new;

CO2_record(round(time/step1))=CO2_new;

O2_record(round(time/step1))=O2_new;

NO3_record(round(time/step1))=NO3_new;

PO4_record(round(time/step1))=PO4_new;

Muave_record(round(time/step1))=Mu_ave;

Iave_record(round(time/step1))=Iave;

% Update the values of X, Glycogen, dissolved CO2 and O2 conventrations

X=X_new;

Glycogen=Glycogen_new;

CO2=CO2_new;

O2=O2_new;

NO3=NO3_new;

PO4=PO4_new;

VCO2_critical=3.7*CO2/(CO2+8/1000);

dCO2=round(VCO2_critical/0.0068398);

VNO3_critical=NO3/X/step1;

dNO3=round(VNO3_critical/0.0014726);

VPO4_critical=0.060955*PO4/(PO4+1/1000);

dPO4=round(VPO4_critical/1.1268097e-04);

Vg_critical=Glycogen/X/step1;

end

% Create a matrix to store values of various parameters

n=200;

step3=numoftime/n;

Results=zeros(n,12);

%Physiological parameters

for k=1:n

j=round(numoftime/n*(k-1)+1);

Results(k,:)=[(j+step3/2)*step1/24,mean(X_record(j:j+step3-1)),mean(Glycogen_record(j:j+step3-1)),mean(CO2_record(j:j+step3-1)),...

mean(O2_record(j:j+step3-1)),mean(Muave_record(j:j+step3-1)),mean(NO3_record(j:j+step3-1)),mean(PO4_record(j:j+step3-1)),mean(I0_record(j:j+step3-1)),...

mean(Population(1,j:j+step3-1)),mean(Population(2,j:j+step3-1)),mean(Population(3,j:j+step3-1))];

end

% Fluxes

ReactionResults=zeros(n,9);

for k=1:n

j=round(numoftime/n*(k-1)+1);

ReactionResults(k+1,:)=[(j+step3/2)*step1/24,mean(R136_record(j:j+step3-1)),mean(R298_record(j:j+step3-1)),mean(R359_record(j:j+step3-1)),...

mean(R464_record(j:j+step3-1)),mean(R502_record(j:j+step3-1)),mean(R549_record(j:j+step3-1)),mean(R554_record(j:j+step3-1)),mean(R585_record(j:j+step3-1))];

end

% Light intensity

I_matrix=zeros(100,100);

for t=1:1:100

for r=1:1:100

X=Results((t-1)*2+1,2);

l=(r-1)/100*radius;

I_matrix(t,r)=I0/((0.0216*l+1)^(1.54))/((0.130*X*l+1)^(1.18));

end

end
